# Metasurface Vision Transformer: A Generic AI Model for Metasurface Inverse Design

**DOI:** 10.1002/nap2.70001

**Published:** 2026-01-13

**Authors:** Jiahao Yan, Jilong Yi, Churong Ma, Yanjun Bao, Qin Chen, Baojun Li

**Affiliations:** ^1^ Guangdong Provincial Key Laboratory of Nanophotonic Manipulation, Institute of Nanophotonics, College of Physics and Optoelectronic Engineering Jinan University Guangzhou China

**Keywords:** artificial intelligence, inverse design, metasurfaces, Vision Transformer

## Abstract

Metasurfaces enable diverse applications by controlling light's amplitude, phase, and polarization. Although deep learning‐based inverse design has revolutionized metasurface design, current models are limited by fixed operating conditions and lack universality, often requiring retraining for new wavelengths, polarizations, or application scenarios. To address this, we introduce MetasurfaceViT (Metasurface Vision Transformer), a generic AI model for inverse design. Our solution leverages a large dataset of Jones matrices, significantly expanded via physics‐informed data augmentation. By pretraining through masking wavelengths and polarization channels, MetasurfaceViT can reconstruct full‐wavelength Jones matrices, which are then used by a fine‐tuning model for inverse design. This versatility allows one‐shot structure design for arbitrary wavelength, polarization, and application requirements. We demonstrate MetasurfaceViT's capabilities in designing multiplexed printings and holograms and broadband achromatic metalenses. Prediction accuracy exceeds 99% for physically realistic designs, showcasing a significant step toward a universal optical inverse design paradigm.

## Graphical abstract

MetasurfaceViT is a generic AI model that addresses limitations in current metasurface inverse design by enabling universal control across wavelengths and polarizations. The authors detail their approach using Jones matrices and physics‐informed data augmentation for full‐wavelength reconstruction, alongside a fine‐tuning methodology. They showcase MetasurfaceViT's capabilities in designing multiplexed printings/holograms and broadband achromatic metalenses, highlighting its high accuracy and transfer learning potential.

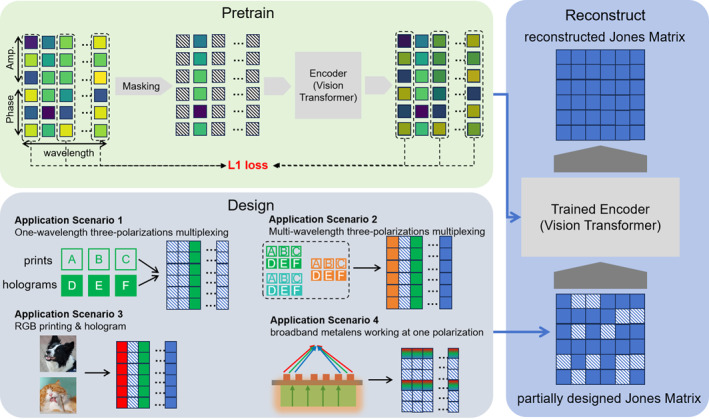

## 1 Introduction

Optical design plays a crucial role in modern technology, as it allows for the precise manipulation of light, facilitating a wide array of applications such as high‐speed communication, high‐resolution imaging, and advanced sensing [1, 2, 3, 4, 5, 6, 7, 8, 9, 10, 11, 12]. In recent years, a revolutionary technique known as optical inverse design has emerged. This approach harnesses the power of deep learning to establish a nonlinear mapping between optical structures and their functional properties [1, 8, 10, 13, 14], circumventing time‐consuming traditional design procedures and achieving higher design accuracy [3, 5]. It has found extensive applications, including the design of nanophotonic devices, metasurfaces for beam steering, polarization holograms, and chiral metasurfaces for biosensing [1, 3, 4, 5, 6, 7]. Meanwhile, the remarkable advancements and rapid iterations in AI models have continuously reshaped the relationship between AI and science. Large language models (LLMs), built on the Transformer framework [15], have attained human‐level intelligence, spurring extensive research on LLM‐based autonomous agents for diverse applications [16]. The Transformer framework has also demonstrated outstanding performance in computer vision [17], protein structure prediction [18], and chemical toxicity assessment [19]. Regardless of whether leveraging LLMs or constructing expert models, AI has profoundly accelerated scientific discovery [20, 21]. As AI models become increasingly sophisticated, the key to enhancing performance has shifted from a model‐centric to a data‐centric approach [22]. Scaling laws indicate that large‐scale data are essential for optimizing model performance and resource utilization [23, 24].

However, current research on metasurface design using deep learning models faces significant limitations. Although considerable progress has been made in enhancing training efficacy and design accuracy, these models typically operate only for fixed wavelengths and polarizations [25, 26, 27]. Although transfer learning and physics‐informed training methods have been successfully proposed to achieve wavelength transfer and improve model versatility [28, 29, 30, 31], a change in input‐output vector size often necessitates retraining, highlighting the lack of universality in these models. On the other hand, current research on metasurface inverse design also suffers from a lack of compatibility across different application scenarios. Researchers have made fruitful efforts to enhance model compatibility, for instance, by utilizing a single physical metric to represent multiple problems [32, 33], improving generality and discovering spectral correlation through physical connections [34], employing powerful LLMs to fine‐tuned optical subtasks [35], or preparing large amounts of data to train an “OptGPT” model [36]. Nevertheless, a versatile and generic design model that can handle diverse applications (encompassing the design of phases and amplitudes for arbitrary wavelengths and polarizations) remains elusive.

In this paper, we introduce MetasurfaceViT, a generic AI model for metasurface inverse design. This paper details MetasurfaceViT's development and application, focusing on its optics‐specific adaptation, training methodology, and demonstrated capabilities. Inspired by the Vision Transformer (ViT) [37], which achieves state‐of‐the‐art performance in computer vision by treating an image as a sequence of patches, we adapt this strategy for optical design. However, our input data consist of optical parameters, specifically Jones matrices [38] at multiple wavelengths, rather than typical image data. These matrices describe how the phase and amplitude of incident light change via interaction with the optical element. Given the relatively small input dimension of Jones matrices, we forgo the traditional patching process. Instead, we treat each physically significant matrix element as an individual token, a computationally efficient approach that is also crucial for preserving distinct optical information at each wavelength. The inherent rich physics within Jones matrices significantly benefits the development of a general‐purpose large model.

We detail our approach through the following key contributions:

– Firstly, through implementing physics‐informed data augmentation, we achieve a cost‐effective data acquisition process and substantially expand the Jones matrices training dataset to 60 million samples.
– Secondly, inspired by language models and masking pretraining in computer vision (CV), we pretrained the model by randomly masking wavelengths and polarization channels. This enables the reconstruction of full‐wavelength Jones matrices from various polarization and wavelength combinations, facilitating subsequent designs.
– Thirdly, based on the pretrained model for Jones matrix reconstruction, we fine‐tuned it to achieve inverse design from the Jones matrix to structural parameters and constructed a forward prediction network to evaluate the performance.
– Moreover, we devised a workflow for constructing predicted units and evaluating metasurface performance as a whole.

Consequently, MetasurfaceViT represents a versatile model/framework in metasurface design, capable of one‐shot structure design for arbitrary wavelength, polarization, and application requirements. We demonstrate MetasurfaceViT's capabilities by successfully performing two classic yet complex metasurface design tasks: multiplexing of printings and holograms, and designing broadband achromatic metalenses. We verified that the prediction accuracy can exceed 99% under physically realistic designed amplitudes and phases and can reach over 85% even for ideal ones. Furthermore, our pretrained models can be efficiently fine‐tuned for new and related optical application scenarios through transfer learning, which significantly reduces the need for extensive new datasets and thereby enhances the model's universality.

## 2 Results

### 2.1 Design of Wavelength‐Dependent Jones Matrix & Masking Strategy

**FIGURE 1:**
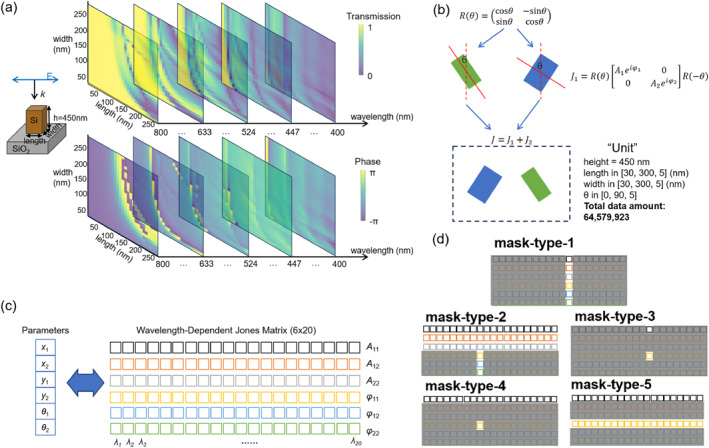
(a) Schematic illustration of the building block of metasurfaces, which consists of Si nanopillars on a $SiO_{2}$ substrate. Presented are a series of transmission and phase distribution maps, demonstrating how variations in the lengths and widths of the Si nanopillars impact them at different wavelengths. (b) Schematic representation of the formation of the “unit cell” in our study through the rotation and pairing of two building blocks. Given the value ranges of widths and lengths, the resultant data size exceeds 60 million. (c) The fundamental structure of the dataset. The input data comprise 20 × 6 Jones matrices, where 20 represents the number of wavelength points and 6 denotes the six independent components (A11, A12, A22, φ11, …) at each wavelength. The labels are the six structural parameters of the “unit.” (d) Schematics depicting the five strategies employed to mask the Jones matrices during the pretraining phase. Mask‐type‐1: retain all six components at a randomly selected wavelength; Mask‐type‐2: preserve amplitudes across all wavelengths while retaining phases at only one randomly chosen wavelength; Mask‐type‐3: maintain only the amplitude and phase of one component at a single wavelength; Mask‐type‐4: keep all amplitudes of one component but preserve the phase of only one component at one wavelength; Mask‐type‐5: retain all amplitudes and phases of one component.

Figure 1 illustrates the key components and strategies in our research on AI‐enabled optical field manipulation. In Figure 1a, we depict the building block of metasurfaces, which consists of a Si nanopillar on a $SiO_{2}$ substrate. The series of transmission and phase distribution maps are crucial, as they reveal the influence of varying lengths and widths of the Si nanopillar on these optical properties at different wavelengths. This information is fundamental for understanding how the physical structure of the metasurface affects light propagation, which serves as the basis for our subsequent data‐driven approaches. Figure 1b shows the concept of constructing a “unit cell” by rotating and pairing up two building blocks.

A Jones matrix, a 2×2 complex matrix, describes the interaction of polarized light with an optical element. It encodes crucial information about both the amplitude and relative phase changes for two orthogonal polarization components at a specific wavelength. Therefore, we utilize Jones matrices to form our dataset, as they inherently capture the full polarization state evolution and phase information essential for accurate and versatile metasurface design [39], which goes beyond what intensity‐only spectral data can provide. As shown in Equation (1), the combined Jones matrix for the unit cell is calculated from rotation‐based matrix multiplication and summation of two building blocks. This combined matrix possesses six degrees of freedom: $A_{11}$, $A_{12}$, $A_{22}$, $\varphi_{11}$, $\varphi_{12}$, and $\varphi_{22}$. Notably, the cross terms ($A_{12}$ and $A_{21}$) are equivalent. The equation presented describes the optical properties at a single wavelength, so the same calculation is repeated across various wavelengths covering the visible range.

(1) $$\begin{array}{cc} J_{\lambda =\lambda n} & =\sum_{i=a\|b}\left[\begin{array}{cc} \cos\theta_{i} & -\sin\theta_{i}\\ \sin\theta_{i} & \cos\theta_{i} \end{array}\right]\\ & \;\times \left[\begin{array}{cc} A_{1i}e^{i\gamma_{1i}} & 0\\ 0 & A_{2i}e^{i\gamma_{2i}} \end{array}\right]\\ & \;\times \left[\begin{array}{cc} \cos\theta_{i} & \sin\theta_{i}\\ -\sin\theta_{i} & \cos\theta_{i} \end{array}\right]\\ & =\left[\begin{array}{cc} A_{11}e^{i\gamma_{11}} & A_{12}e^{i\gamma_{12}}\\ A_{21}e^{i\gamma_{21}} & A_{22}e^{i\gamma_{22}} \end{array}\right] \end{array}$$

By leveraging Jones matrices and corresponding matrix manipulations, we achieve physics‐informed data augmentation. Notably, our physics‐informed mechanism operates at the data preparation stage, distinct from physics‐informed neural networks (PINNs), where physical laws are embedded during model training. This process allows us to expand a dataset of fewer than 1000 samples, initially considering only the width and length of a single block, into millions of data points encompassing the width, length, and angle of two blocks. The generation of the final dataset (size: 64,579,923) via the combination of the two blocks' parameters is detailed in Supporting Information S1: S1.

Therefore, the large data size, exceeding 60 million, is a result of the wide value ranges of widths, lengths, and angles considered. This large dataset provides a rich source of information for training our models, enabling us to explore a vast parameter space and potentially discover novel optical regulation phenomena. As the optical parameters of the two‐block “unit” are derived from Jones matrix‐based calculations, Supporting Information S1: S2 presents comprehensive spectral visualizations and statistical analyses to verify their consistency with the direct simulation data of the two‐block system.

The dataset structure is presented in Figure 1c. We use 20×6 Jones matrices as input data, where the 20 represents the number of wavelength points in the visible‐light region, and the 6 corresponds to the three amplitude components and three phase components of the Jones matrix at each wavelength. The six structural parameters of the “unit” are used as labels. This mapping between the input data and labels is the cornerstone of our training process, as it allows our models to learn the relationship between the physical structure and the optical response.

Figure 1d details the five masking strategies employed during the pretraining phase. These strategies are designed to enhance the generalization ability of our models. To avoid oversampling or undersampling in specific cases, mask types were randomly selected during training. Additionally, for mask types involving wavelength selection, wavelength channels were randomly chosen. This random selection of mask types and wavelengths ensures that the model can uniformly handle most application scenarios operating at arbitrary wavelengths. Careful design of the masking types enables the model to learn implicit optical principles during training. For example:

– Mask‐type‐1 retains all six components (e.g., A11, A12, A22, φ11 …) of the Jones matrix at a single random wavelength, allowing the model to independently learn the characteristics of individual wavelengths while disregarding others. It is particularly suited for metasurface designs targeting single‐wavelength polarization and amplitude modulation.
– Mask‐type‐2 preserves amplitude components across all wavelengths while retaining phase components at only one random wavelength. It is tailored for complex multi‐wavelength multiplexing scenarios, where the phase at target wavelengths must satisfy imaging or holographic requirements, and full‐band amplitude control is necessary to enhance amplitude at specific bands while suppressing it at others—thus highlighting the importance of amplitude information.
– Mask‐type‐3 retains only the amplitude and phase of a specific polarization channel at a single wavelength, corresponding to the simplest design scenario. However, as it provides the least information and requires the model to reconstruct the most details, it is the most challenging masking type for training—compelling the model to focus on specific dimensions of the optical response.
– Mask‐type‐4 bears a relational similarity to Mask‐type‐3 analogous to that between Mask‐type‐1 and Mask‐type‐2 but exclusively focuses on a single polarization channel. Specifically, it retains the full‐wavelength amplitude and single‐wavelength phase of the target polarization channel, facilitating the implementation of multi‐wavelength multiplexing designs.
– Mask‐type‐5 preserves both amplitude and phase components of a single polarization channel across all wavelengths. It is primarily designed to train the model in Jones matrix reconstruction for broadband metalenses, further refining the model's focus on the broadband characteristics of specific polarization channels.

### 2.2 Workflow of Pretraining, Design, and Reconstruction

**FIGURE 2:**
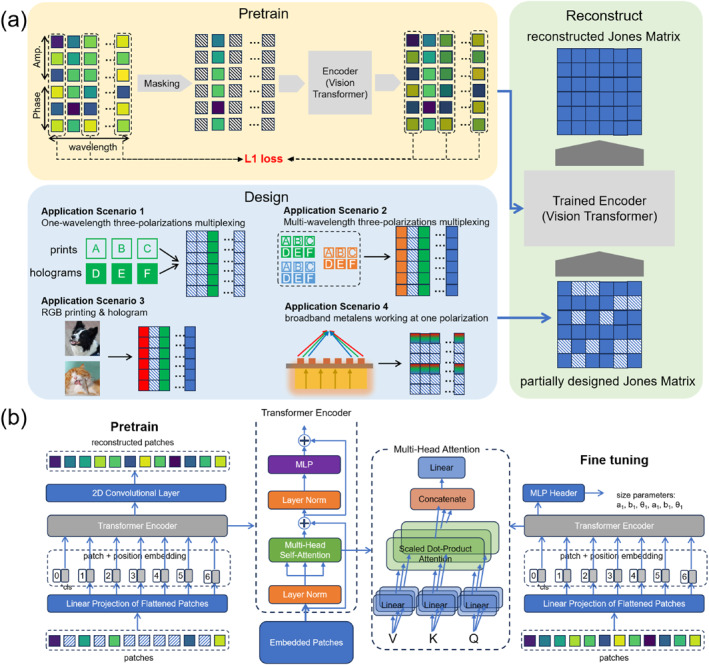
(a) Workflow of the pretraining, design, and reconstruction phases. Pretraining: Jones matrices from the training set are masked using different masking strategies and then input into the ViT network. The network is trained by minimizing the L1 loss between the generated values and the actual values of the masked elements. Design: Generate the corresponding Jones matrix based on diverse application scenarios and populate the 20 × 6 full‐scale Jones matrices. As long as an application scenario can be manifested and represented by at least one “pixel” within the 20 × 6 Jones matrices, it can be addressed. Typical application scenarios include three‐polarization multiplexing at single or multiple wavelengths, RGB holograms, routing or printing, and broadband metalenses. Reconstruction: Utilize the incomplete Jones matrices from the design phase and feed them into the pretrained ViT network. Eventually, complete Jones matrices with filled blank elements are obtained. (b) Architecture of the ViT network during the pretraining and fine‐tuning phases.

Figure 2 illustrates the workflow and architecture of a method that combines AI and optics, specifically focusing on the use of a ViT in the context of Jones matrix manipulation. The rationale for employing a ViT over other AI models lies in its powerful masking pretraining mechanism [40]. This mechanism is uniquely suited for reconstructing full‐wavelength optical data due to the ViT's unparalleled ability to capture long‐range dependencies and intricate patterns within sequential or spatially distributed data. Unlike variational autoencoders (VAEs) that often excel at generating diverse but potentially less precise samples, the ViT's attention mechanism enables a highly accurate and context‐aware reconstruction of missing optical parameters, which is critical for the high fidelity required in inverse design problems. Even with integrated attention layers, VAEs retain a core focus on generating diverse samples rather than precisely recovering masked optical parameters—distinct from the ViT's masking pretraining, which is inherently tailored for Jones matrix reconstruction. Furthermore, VAEs' latent space compression weakens fine‐grained optical correlations across wavelengths and polarizations, whereas the ViT's self‐attention directly models these long‐range dependencies.

In the pretraining phase, as depicted in the upper‐left part of Figure 2a, Jones matrices from the training set are masked using various masking strategies. These masked matrices are then fed into the ViT network. The goal of this training process is to minimize the L1 loss between the generated values and the actual values of the masked elements. The L1 loss, also known as mean absolute error (MAE), is described by Equation (2):

(2) $$L=\frac{1}{\Omega \left(x_{\mathrm{mask}}\right)}{\left\|y_{\mathrm{mask}}-x_{mask}\right\|}_{1}$$

where $x_{\mathrm{mask}}$ represents the actual values of the masked elements in the input Jones matrix, $y_{\mathrm{mask}}$ denotes the predicted values for those masked elements, and $\Omega \left(x_{\mathrm{mask}}\right)$ is the number of masked elements. This optimization step through gradient descent is crucial for training the network to accurately predict the masked parts of the Jones matrices.

The design phase, as shown in the middle section of Figure 2a, maps user requirements for metasurfaces to Jones matrices. Different application scenarios [38, 39, 41], such as one‐wavelength three‐polarization multiplexing, multi‐wavelength three‐polarization multiplexing, RGB printing and holograms, and broadband metalens working at one polarization, are considered. Specifically, the process for obtaining phase distributions based on target hologram images is detailed in Supporting Information S1: S3. This iterative workflow is based on the well‐established Gerchberg–Saxton algorithm [42]. The corresponding Jones matrices are generated and partially filled into a 20×6 full‐scale Jones matrix. This process allows for the representation of diverse optical applications within a unified matrix framework. The selection of multiplexing of printings and holograms and broadband achromatic metalenses as example applications in this study is because of their demanding requirements for simultaneous control over multiple optical properties across varying wavelengths and polarizations. These applications necessitate precise manipulation of amplitude, phase, and polarization responses, making them ideal benchmarks for a versatile inverse design framework. The reconstruction phase, as shown in the upper‐right part of Figure 2a, takes the partially filled Jones matrices from the design phase and passes them through the pretrained ViT network. The network then reconstructs the blank elements, resulting in a complete Jones matrix. This complete matrix is essential for subsequent processing in the fine‐tuning workflow.

Figure 2b presents the architecture of the ViT network during the pretraining and fine‐tuning phases. In the pretraining phase, the network processes reconstructed patches through a series of operations, including a 2D convolutional layer, a linear projection of flattened patches, and the ViT encoder itself. As discussed above, our ViT model skipped the patching process, so each patch actually means each component in the Jones matrices. To be consistent with mainstream analysis of the ViT framework, we still use the term “patch” in our analysis. The ViT encoder consists of a multihead self‐attention mechanism, layer normalization, and a multilayer perceptron (MLP). The multihead self‐attention mechanism is a core component that enables the model to simultaneously focus on different parts of the input sequence. It achieves this by transforming input embeddings into query (Q), key (K), and value (V) vectors and then computing attention scores via dot products between Q and K. This mechanism allows for the capture of diverse relationships and long‐range dependencies within the optical data. The hyperparameter tuning process for the pretraining phase, including batch size, data size, and learning rate, is detailed in Supporting Information S1: S4, confirming that the current set of parameters is optimized.

The impact of model parameter count on training efficiency and performance, along with a comparison between the original 37.7M‐parameter model and a lightweight 4.7M‐parameter variant (exhibiting reduced training time but higher loss, with comparable inference speed), is detailed in Supporting Information S1: S5. In addition, a detailed analysis of the patching strategy's impact and a corresponding learning curve comparison is provided in Supporting Information S1: S6.

During the fine‐tuning phase, the network output is adjusted to generate size parameters. The key difference between the pretraining and fine‐tuning phases lies in the output of the network. Although the pretraining phase focuses on reconstructing the Jones matrix, the fine‐tuning phase aims to optimize specific parameters related to the application. This combination of pretraining, design, reconstruction, and fine‐tuning processes using a ViT network in the context of Jones matrices provides a powerful framework for solving various optical problems. Our ViT model comprises 37,703,489 learnable parameters, primarily distributed across the patch embedding, multihead self‐attention, and feed‐forward layers. This substantial parameter count is crucial for enabling the model to learn the complex, long‐range dependencies inherent in optical data. Critically, with over 60,000,000 data samples, our dataset is of a magnitude comparable to large‐scale computer vision corpora such as ImageNet or LSUN, providing a robust training signal that effectively prevents overfitting and allows the model to fully utilize its capacity without underfitting [37, 40].

### 2.3 Workflow of Fine‐tuning, Prediction, and Evaluation

**FIGURE 3:**
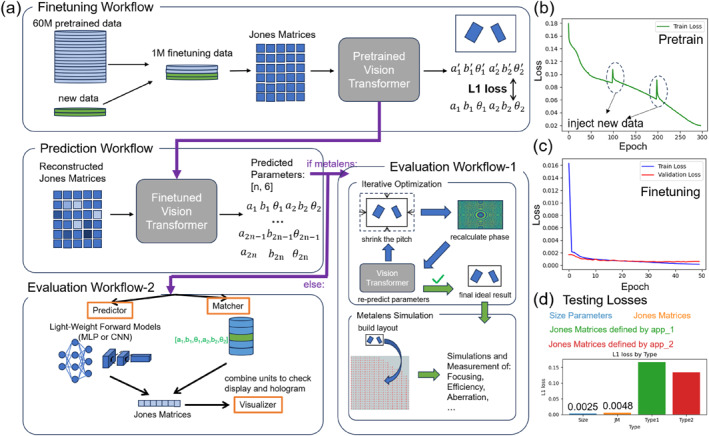
(a) Workflows of the fine‐tuning, prediction, and evaluation phases. When the design type is a metalens, Evaluation Workflow‐1 is initiated. This workflow encompasses iterative size and pitch generation, followed by FDTD‐based metalens simulation. In other scenarios, Evaluation Workflow‐2 is activated, which consists of a predictor, a matcher, and a visualizer. (b) The epoch‐dependent L1 loss during the pretraining phase. Notably, the two peaks observed at epochs 100 and 200 result from the data splitting and distributed injection techniques implemented to circumvent memory overhead. (c) The epoch‐dependent L1 loss during the fine‐tuning phase. Given that the fine‐tuning task is considerably less complex than pretraining, the model can attain a very low loss after only 50 epochs. (d) Evaluation of Jones matrices (JM) from the test set and designed Jones matrices. “Size”: represents the loss of the inverse design process (JM → size parameters). “JM”: denotes the loss of the tandem workflow (JM → size parameters → JM). “Type 1”: corresponds to the L1 loss between the designed JM (not for metalens) and the predicted JM through the tandem workflow. “Type 2”: signifies the L1 loss between the designed JM (specified for metalens) and the predicted JM through the tandem workflow.

Figure 3 illustrates the workflows of the fine‐tuning, prediction, and evaluation phases, along with the associated loss metrics during different training and testing stages. The fine‐tuning workflow (Figure 3a) starts with a combination of 60M pretrained data and new data, which are randomly sampled to form a 1M fine‐tuning dataset. The pretrained ViT serves as the starting point, and the fine‐tuning process adjusts the network weights. Unlike the pretraining phase, the loss function in fine‐tuning is the L1 loss between the target and generated structural parameters. This approach allows the model to adapt more precisely to the specific requirements of the new data related to Jones matrices.

In the prediction workflow, reconstructed Jones matrices, generated using the pretrained network, are fed into the fine‐tuned ViT. The output is a set of n×6 structural parameters, where *n* represents the number of structures. The remarkable speed of this generation, such as for a 256×256 metasurface with *n* = 65,536, which can be completed in seconds, showcases the efficiency of the model. The evaluation workflow is crucial for assessing the rationality of the generated structural parameters. It can be seen as a forward‐prediction process, simulating optical effects before the experimental fabrication of metasurfaces. Evaluation Workflow‐1 is specifically designed for metalenses, which involves iterative optimization due to the sensitivity of metalens phase design to the absolute position of units and the need to reduce the pitch between units for efficiency. By iteratively shrinking the pitch and recalculating the phase distribution, the process aims to achieve an optimal design while avoiding excessive fabrication difficulty (with a minimum gap of 100 nm). Evaluation Workflow‐2, on the other hand, is for conventional metasurfaces, using a predictor (a lightweight trained network) and a matcher (searching in pretrained data) to map structural parameters to Jones matrices, followed by visualization steps.

In Figure 3b, the epoch‐dependent L1 loss during the pretraining phase is shown. The two peaks at epochs 100 and 200 are due to data splitting and distributed injection to manage memory overhead. This indicates the challenges in handling large‐scale data during pretraining. Figure 3c depicts the epoch‐dependent L1 loss during the fine‐tuning phase. The model can achieve a very low loss after 50 epochs, highlighting that the fine‐tuning task is relatively easier than pretraining because the model already has a good initial set of weights from pretraining. In Supporting Information S1: S7, we present the fine‐tuning learning curve without the benefit of pretrained weights. This further demonstrates that fine‐tuning based on a pretrained model significantly accelerates convergence and enhances accuracy. Moreover, the importance of pretraining is underscored by its masking mechanism, which is essential for the reconstruction phase, as discussed previously.

Figure 3d evaluates the performance on Jones matrices from the test set and designed Jones matrices. The low L1 loss of 0.0025 for the inverse design (Jones matrices → size parameters) and 0.0048 for the tandem workflow (Jones matrices → size parameters → Jones matrices) on the test set demonstrates the effectiveness of the model. However, the higher losses for designed Jones matrices (approximately 0.15 for non‐metalens and 0.13 for metalens) suggest that the ideal designed matrices may not be fully realizable in practice, as they represent an overly simplified or theoretical scenario. The iterative and efficient nature of the workflows, along with the analysis of loss metrics, contributes to the understanding of how the model can be optimized for practical applications in metasurface design while also highlighting the challenges in bridging the gap between theoretical designs and real‐world implementation.

### 2.4 Example Application 1: Multiplexing of Printings and Holograms

**FIGURE 4:**
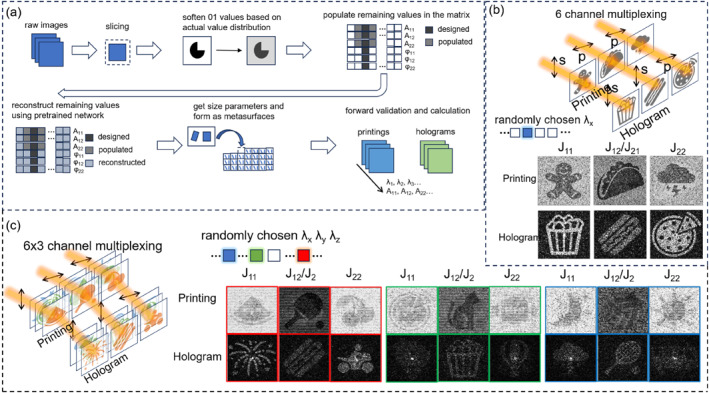
(a) Schematic illustration of the process for designing and evaluating multiplexing metasurfaces. (b) Demonstration of 6‐channel printing and hologram multiplexing achieved at a single wavelength. (c) Illustration of 18‐channel printing and hologram multiplexing accomplished at three wavelengths.

Figure 4 illustrates the workflow for designing metasurfaces for multiplexing printings and holograms, as well as the example results of single‐ and multi‐wavelength multiplexing. In Figure 4a, the process begins with raw images, which are sliced to obtain target images of appropriate sizes. The binary values in the images are then softened based on the actual value distribution. For example, the values of 0 and 1 are adjusted according to the amplitude distribution at the corresponding wavelength to ensure that the designed Jones matrix conforms to the actual values. After designing the amplitude and phase for a certain wavelength, the values of other relevant wavelengths need to be populated. For instance, when designing the red part of the RGB channels, the amplitude of the red part is kept normal, whereas the blue and green parts are assigned minimum values to minimize the interference of the red unit on other channels. The remaining part of the Jones matrix is reconstructed using the pretrained network, and then the size information of the unit is obtained through a fine‐tuned network. This operation is performed on each unit of the metasurface, and the size information is used to generate a predicted Jones matrix through a forward network. The amplitude and phase are calculated according to the corresponding components to evaluate the effects of printings and holograms.

Figure 4b shows the six‐channel printing and hologram multiplexing at one wavelength. By encoding information in A11, A12, A22, φ11, φ12, and φ22, six‐channel information multiplexing can be achieved. As shown in the schematic, by changing the polarization directions of the polarizer and analyzer, multiple channels of information can be read out. The resulting printings and holograms demonstrate good performance, highlighting the flexibility of the model in reverse designing at any selected wavelength.

Figure 4c presents the 18‐channel printing and hologram multiplexing at three wavelengths. Similar to the single‐channel case, six‐component information multiplexing is achieved at each of the three randomly selected wavelengths. Although the 6 × 3 channel multiplexing is basically achieved, some holograms suffer from high noise levels, making it difficult to clearly identify the images. This is due to the idealized design that cannot be fully reproduced, such as the overlapping of the designed hologram images in the three channels, which causes crosstalk. The design performance can be improved by selecting wavelength channels that are further separated, thereby minimizing crosstalk. As demonstrated in Supporting Information S1: S8, this approach yielded better printings and holograms. Furthermore, in Supporting Information S1: S8, we delve deeper into strategies for minimizing unrealistic constraints during the design phase by iteratively minimizing the differences between designed and reconstructed Jones matrices. The previous loss information has proven that accurate design can be achieved as long as the designed Jones matrix is close to physical reality.

### 2.5 Example Application 2: Broadband Achromatic Metalens

**FIGURE 5:**
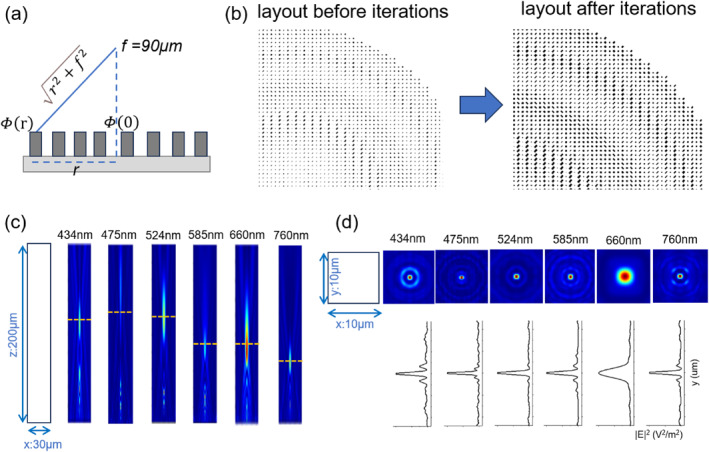
(a) Schematic representation of the calculation methodology for phase distribution. (b) Layouts of the metalens before and after iterative optimization, utilized to enhance the inter‐unit distance. (c) Normalized electric field profiles within the *x*–*z* plane, indicative of the focusing length corresponding to each wavelength. (d) Normalized electric field profiles in the *x*–*y* plane and line‐scan electric field intensities along the *y*‐axis, illustrating the focusing performance at each wavelength.

Figure 5 is used to evaluate the broadband achromatic metalens designed through our proposed model. In Figure 5a, a schematic illustration shows the method of calculating the phase distribution of each unit in the metalens. The phase calculation is based on the distance of each unit from the origin (r) and the designed focal length (f=90μm). This approach is fundamental for achieving the desired optical performance of the metalens. Conventional methods for optimizing broadband achromatic metalenses typically require satisfying not only spatially and frequency‐based phase profiles but also considering the group delay and group delay dispersion, as indicated by the Taylor expansion in Equation (3):

(3) $$\begin{array}{cc} \varphi \left(r,\omega \right) & =\varphi \left(r,\omega_{d}\right)+{\left.\frac{\partial \varphi }{\partial \omega }\right|}_{\omega =\omega_{d}}\left(\omega -\omega_{d}\right)\\ & +{\left.\frac{\partial^{2}\varphi }{2\partial \omega^{2}}\right|}_{\omega =\omega_{d}}{\left(\omega -\omega_{d}\right)}^{2}+\text{…} \end{array}$$

By employing our one‐shot inverse design approach, we can circumvent this sophisticated optimization process. Figure 5b presents the layouts of the metalens before and after iterative optimization. The initial layout, before iterations, shows a relatively sparse arrangement of metalens units, which is less favorable for interaction with the light field. In contrast, the layout after iterations is more densely packed while still achieving the preset phase distribution. This demonstrates the significance of the iterative algorithm in optimizing the distance between units, enhancing the efficiency of light manipulation.

Figure 5c displays the normalized electric field profiles at the *x*–*z* plane, which indicate the focusing performance of the metalens across six representative wavelengths in the visible‐light spectrum (434, 475, 524, 585, 660, and 760 nm). The 3D simulation, though time‐consuming, accurately represents the light field. The focusing length is approximately 90μm as designed, but a chromatic effect is observed, where the focusing length decreases with an increase in wavelength. This chromatic aberration is an area for improvement. Figure 5d shows the normalized electric field profiles at the *x*–*y* plane and the line‐scan electric field intensities along the *y*‐axis. It reveals that the metalens has good focusing performance at all six wavelengths, with the full width at half maximum (FWHM) generally within 1 μm, meeting the basic practical requirements. Regarding the less‐than‐ideal achromatic performance, it is mainly attributed to the strong amplitude constraints during the design phase. When all wavelength amplitude components are set to the maximum value and the phase distribution is applied to all phase components, it may sacrifice some phase constraints. This can lead to deviations in the actual predicted Jones matrix.

To address this issue, we explored two alternative design strategies in Supporting Information S1: S9. First, we masked all amplitudes instead of fixing them to their maximum values. This strategy allows the reconstruction model to freely generate amplitude components, prioritizing phase fidelity. Although this approach improves achromatic performance (focusing length standard deviation reduced from 20.9 to 7.2 nm), focusing efficiency degrades due to the relaxed amplitude constraint. As proposed in Example Application 1, metalens design accuracy can be further improved by iterative optimization to align target Jones matrices with physical constraints. Detailed implementation of gradient descent‐based iterative optimization is provided in Supporting Information S1: S5. Results demonstrate achromatic performance comparable to the amplitude‐masking strategy (focusing length standard deviation: 9.3 nm), whereas field distributions across wavelengths indicate superior focusing efficiency relative to the amplitude‐masking approach. Although the iterative method outperforms both the original non‐masking and amplitude‐masking approaches, its runtime is hundreds of times longer than one‐shot inverse design, indicating a trade‐off between efficiency and accuracy. Because of the inherent limitations of silicon as a building block for visible‐light achromatic metalenses—including low transmission efficiency from strong wavelength‐dependent absorption and inevitable intensity chromatic aberration—none of the explored strategies achieve fully satisfactory performance. This necessitates the adoption of $TiO_{2}$‐based systems in future research, which can be realized via transfer learning using the proposed model.

## 3 Discussion

Our model demonstrates versatility in handling optical design requirements across various wavelengths and polarization states. Besides the two example applications presented above, our model is also capable of designing functional metasurfaces—including those that manipulate circular/elliptical polarization states, generate vortex beams, achieve broadband beam splitters, and produce vivid structural colors. As shown in Figure 2a, as long as desired optical properties are encoded at specific wavelength(s) or polarization(s), the subsequent reconstruction and prediction workflows remain identical to the examples presented. For optical properties beyond the scope of predefined Jones matrices, to further extend its applicability, we show in Supporting Information S1: S7 that the model can be readily transferred to different wavelength ranges and pillar heights using only hundreds of new data points. Through physics‐informed data augmentation and a few‐epoch fine‐tuning process, the prediction accuracies for these new tasks are comparable to those achieved for the default tasks leveraging the pretraining data. In Supporting Information S1: S10, a table comparing recent optical inverse design papers is presented to highlight our work's novelty in expanding model universality. This table details the input/output types of these models, whether target wavelengths/polarizations are tunable, and whether our model can reproduce their use cases. We observe that our model can achieve 7 out of 9 use cases from these studies; for the remaining uncovered use cases, we can leverage the aforementioned transfer learning to achieve them easily.

It is also important to clarify the distinct advantages of inverse design over iterative forward design for optical tasks. Inverse design offers significant benefits in optical systems by directly generating structural parameters from a desired optical spectrum, enabling rapid, “one‐shot” solutions. This approach can explore a much broader and often nonintuitive design space, leading to novel and high‐performance devices that traditional, intuition‐driven forward methods might miss. However, a potential drawback of inverse design is that ideal or unrealistic target optical responses might lead to higher deviations in a one‐shot process compared to a cautious iterative approach.

We further compare data retrieval methods with our deep learning‐based prediction approach in Supporting Information S1: S11, demonstrating that the neural network not only exhibits superior generalization to unseen target Jones matrices (unachievable by retrieval methods) but also offers dramatically improved efficiency—whileclarifying the role and limitations of the “matcher” step in post‐training evaluation.

It is also necessary to explain in depth why ViT is the ideal choice for the proposed application scenarios. Firstly, and most importantly, the ViT's masking pretraining mechanism allows for the reconstruction of full‐scale Jones matrices from arbitrary masked portions. This capability is crucial for predicting diverse optical requirements, as it enables the model to handle varying input‐output vector sizes and reconstruct comprehensive optical responses, circumventing the need for retraining with every change. Beyond this, ViTs excel at capturing long‐range dependencies across spatial dimensions, analogous to how they process relationships between different patches in an image. Therefore, this architecture can effectively learn complex correlations between various wavelengths, polarizations, and the resulting optical properties within the Jones matrices. Empirically, ViTs have demonstrated remarkable performance in tasks involving large‐scale data. Their scalability and ability to leverage extensive data through self‐attention mechanisms are key to optimizing model performance and achieving the goal of a “generic model” for optical design.

## 4 Conclusion

In conclusion, MetasurfaceViT emerges as a game‐changing force in metasurface inverse design. By enlarging the dataset to 60 million samples and introducing Jones matrices and physics‐informed augmentation, we found a cost‐effective way to build a large AI model in optics. The novel pretraining approach, involving random masking of wavelengths and polarization channels, empowered the model to handle diverse combinations adeptly. Fine‐tuning it for the transition from the Jones matrix to structural parameters and building a forward prediction network further enhances its capabilities. Our verification on two typical application scenarios, namely, the multiplexing of printings and holograms and the design of broadband achromatic metalenses, confirmes remarkable prediction accuracies, surpassing 99% under realistic conditions and reaching over 85% for ideal ones. We also discuss two key areas for future improvement of our model. One is the application of transfer learning, utilizing smaller amounts of data for fine‐tuning to address an even wider array of application scenarios (e.g., different pillar heights and varying working wavelengths). The other involves leveraging the reconstruction workflow to iteratively optimize the “input optical prompts.” This approach aims to create physics‐constrained designs, which can yield more precise reconstructed Jones matrices and, consequently, higher prediction accuracy in subsequent workflows. MetasurfaceViT thus paves the way for efficient, one‐shot metasurface structure design, fulfilling arbitrary wavelength, polarization, and application demands, heralding a new era in this domain.

## 5 Experimental Section

### 5.1 Code Availability

The code used in this research is publicly available on GitHub at https://github.com/JYJiahaoYan/MetasurfaceVIT. This repository encompasses all the code related to AI and optical calculations employed in the study. It is structured into several key components: data generation, masked pretraining, metasurface design, Jones matrix reconstruction, fine‐tuning of pretrained models, parameter prediction, and metasurface forward prediction.

### 5.2 Deep Learning Model

The training process was carried out on a workstation equipped with four NVIDIA 4090 GPUs, leveraging the PyTorch distributed training module for enhanced efficiency. Each GPU was assigned a batch size of 128, and consequently, with four GPUs operating in parallel, the overall batch size amounted to 512. When dealing with data on the scale of approximately 60 million samples, one epoch of training took roughly 1 h. For a pretraining phase involving 300 epochs, the estimated time consumption was close to 12.5 days, which fell within an acceptable range. During the fine‐tuning stage, due to the substantially reduced data volume and the requirement of only 50 epochs, the entire fine‐tuning process took approximately 5 h. Notably, the fine‐tuning operation could also be performed on an ordinary computer, which significantly broadened the practical applicability of the model. This study was based on the ViT architecture and incorporated the standard multihead self‐attention mechanism, with the number of heads set to 12 to capture feature information from diverse dimensions. In the model training process, the AdamW optimizer was employed, with the initial learning rate set to 5e‐4 and the weight decay set to 0.05 to effectively prevent overfitting. Meanwhile, a cosine annealing learning rate scheduler was utilized to dynamically adjust the learning rate and facilitate model convergence.

### 5.3 Optical‐Related Calculation and Simulation

Firstly, FDTD simulations were employed to gather the phase and transmission data of single silicon nanopillars as their sizes varied. Then, the Jones matrix for a single structure was built using the amplitude and phase under two orthogonal polarizations, and through matrix operations, the Jones matrices for two rotated nanopillars were obtained. This construction was performed across all wavelength points, ultimately yielding a 20×6 2D matrix as training data (20 for wavelength points and 6 for 3 amplitude and 3 phase components). In the design workflow, target images for printings were directly converted to NumPy arrays via PIL methods. For phase design, the Gerchberg–Saxton algorithm was used for iterative optimization via multiple Fourier transforms and inverse Fourier transforms to derive the phase distribution on the metasurface exit plane from target hologram images. For Evaluation Workflow‐1 (metalens assessment), a 3D metasurface was constructed with FDTD simulations. Array truncation was preapplied to ensure a 30‐μm metalens diameter, and only one quadrant was built by leveraging symmetry to save computation. The far‐field projection and focusing effects were evaluated via direct simulation for higher accuracy despite longer runtime. For Evaluation Workflow‐2 (multiplexed metasurface assessment), when multiple wavelength channels were involved, NumPy arrays were split and recombined to extract wavelength‐specific images from the Jones matrix. Holograms were generated using the fast Fourier transform.

## Author Contributions

All authors have accepted responsibility for the entire content of this manuscript and approved its submission.

## Funding

The work was supported by the Guangdong Basic and Applied Basic Research Foundation (Grant 2023B1515020046) and the National Natural Science Foundation of China (Grant 62475100).

## Conflicts of Interest

The authors declare no conflicts of interest.

## Supporting information


Supporting Information S1

## Data Availability

The datasets generated during and/or analyzed during this study are available from the corresponding author upon reasonable request.
