# Supplementary material for: Metasurface Vision Transformer: A Generic AI Model for Metasurface Inverse Design
Source: Nanophotonics. 2026 Jan 13;15(1):e70001. doi: 10.1002/nap2.70001 (PMC12962286; doi:10.1002/nap2.70001)
Supplement: Supplementary file 1 — Supporting Information S1 [file NAP2-15-e70001-s001.pdf]

Supporting Information

Metasurface Vision Transformer: A Generic AI Model for Metasurface Inverse Design

Jiahao Yan Jilong Yi Churong Ma Yanjun Bao Qin Chen Baojun Li**

Jiahao Yan, Jilong Yi, Churong Ma, Yanjun Bao, Qin Chen, Baojun Li
Guangdong Provincial Key Laboratory of Nanophotonic Manipulation,
Institute of Nanophotonics,
College of Physics and Optoelectronic Engineering,
Jinan University,
Guangzhou 511443, China
Email Address: jhyan@jnu.edu.cn & baojunli@jnu.edu.cn

Keywords: *Metasurfaces, Inverse Design, Artificial Intelligence, Vision Transformer*

1 Details on Data Augmentation

We achieve data augmentation by extending from single nanopillars to paired nanopillars via Jones Matrices and corresponding matrix manipulations. As shown in Figure S1, with the inclusion of rotation, the dataset size for single nanopillars is 6,561. Values formatted as [30, 300, 10] denote [start value, exclusive end value, step size]. When combining pillars A and B, the number of combinations is not 6,561 $\times$ 6,561, as this includes duplicates: placing pillar A on the left and pillar B on the right is equivalent to placing B on the left and A on the right. Therefore, as shown in Figure S1, we only retain data points in the top-left and diagonal regions of the data space, resulting in a dataset size of 21,526,641. Finally, as indicated in Figure 3b (main text), we injected three batches of data at different training epochs to reduce memory storage overhead. The total dataset size is thus 3 $\times$ 21,526,641 = 64,579,923.

2 Comparison between Simulated and Calculated Optical Properties

To validate the consistency between simulated and calculated Jones Matrices of paired nanopillars, we first randomly selected 50 samples (each consisting of a Jones Matrix and corresponding size parameters) from the large dataset. We then used the selected size parameters to construct two nanopillars in FDTD Solutions, computed the scattering parameters, and subsequently mapped them to the corresponding Jones Matrix components for comparison. From the typical spectral comparisons of the A11, A22, φ11, and φ22 components in Figure S2, the calculated and simulated results are in good agreement, particularly for phase components. The statistical results for the 50 samples (Table S1) align with the observations from Figure S2. However, discrepancies in amplitude components become noticeable at wavelengths below 500 nm, as the calculated data relies on linear superposition and does not fully capture all electromagnetic interactions between the two pillars. In contrast, direct FDTD simulation of the paired structure fully captures all electromagnetic interactions (even weak ones), which become more pronounced and complex at shorter wavelengths. Given the consistent pattern of these discrepancies, a specialized machine learning model could be trained to learn the underlying transformation between simulated and calculated Jones Matrices. This approach not only provides a means to reconcile these differences but also holds promise for future transfer learning applications, such as mapping experimental optical data to calculated Jones Matrices.

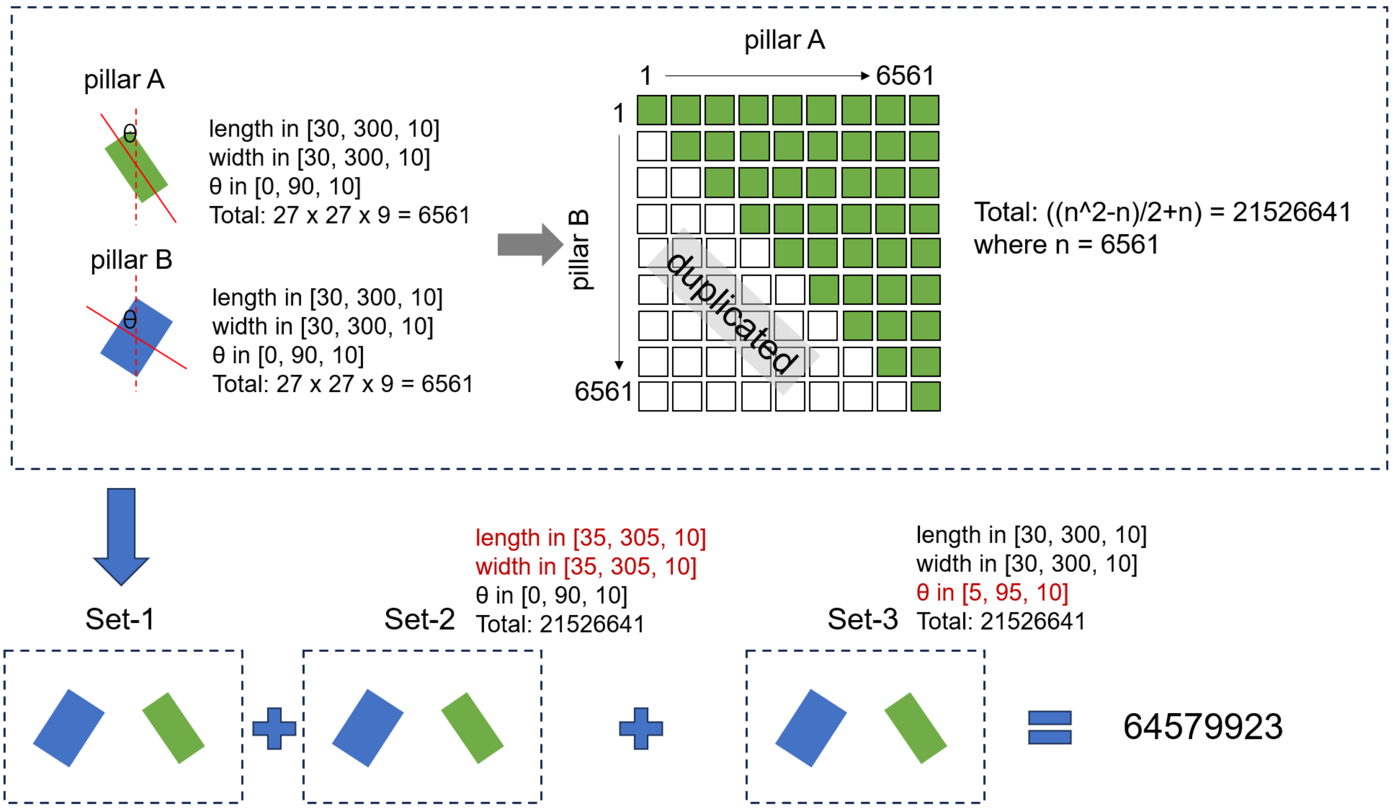

Figure S1: Schematic showing the data size calculation from single to double nanopillars.

Table S1: Mean Squared Error (MSE) and Standard Deviation (STD) of Jones Matrix Parameters Across Wavelengths

Jones Matrix Parameter	MSE_global	STD_global	MSE_best_wavelength	MSE_worst_wavelength
A11	0.069	0.230	0.020	0.132
A22	0.081	0.251	0.022	0.152
φ11	0.018	0.135	0.008	0.066
φ22	0.024	0.156	0.009	0.073

3 Gerchberg–Saxton Algorithm for Hologram Design

To obtain the desired phase distribution that can generate a target hologram image, we employed an iterative optimization process based on the Gerchberg–Saxton (GS) algorithm. The workflow is illustrated in Figure S3. This algorithm iteratively refines the phase distribution by propagating the optical field between two planes: the source plane (target printing intensity) and the hologram plane (target hologram image). Starting with a random phase and the known source intensity, a Fourier Transform is applied to propagate the field to the hologram plane. At this plane, the amplitude is replaced by the target hologram amplitude, while the phase is retained. Subsequently, an Inverse Fourier Transform propagates the field back to the source plane. Here, the amplitude is replaced by the source intensity, and the phase is again retained. This iterative process continues, minimizing the difference between the calculated hologram intensity and the target hologram intensity, until the error converges below a predefined tolerance. The final phase distribution on the source plane is then extracted and discretized to a specified number of phase levels for practical implementation.

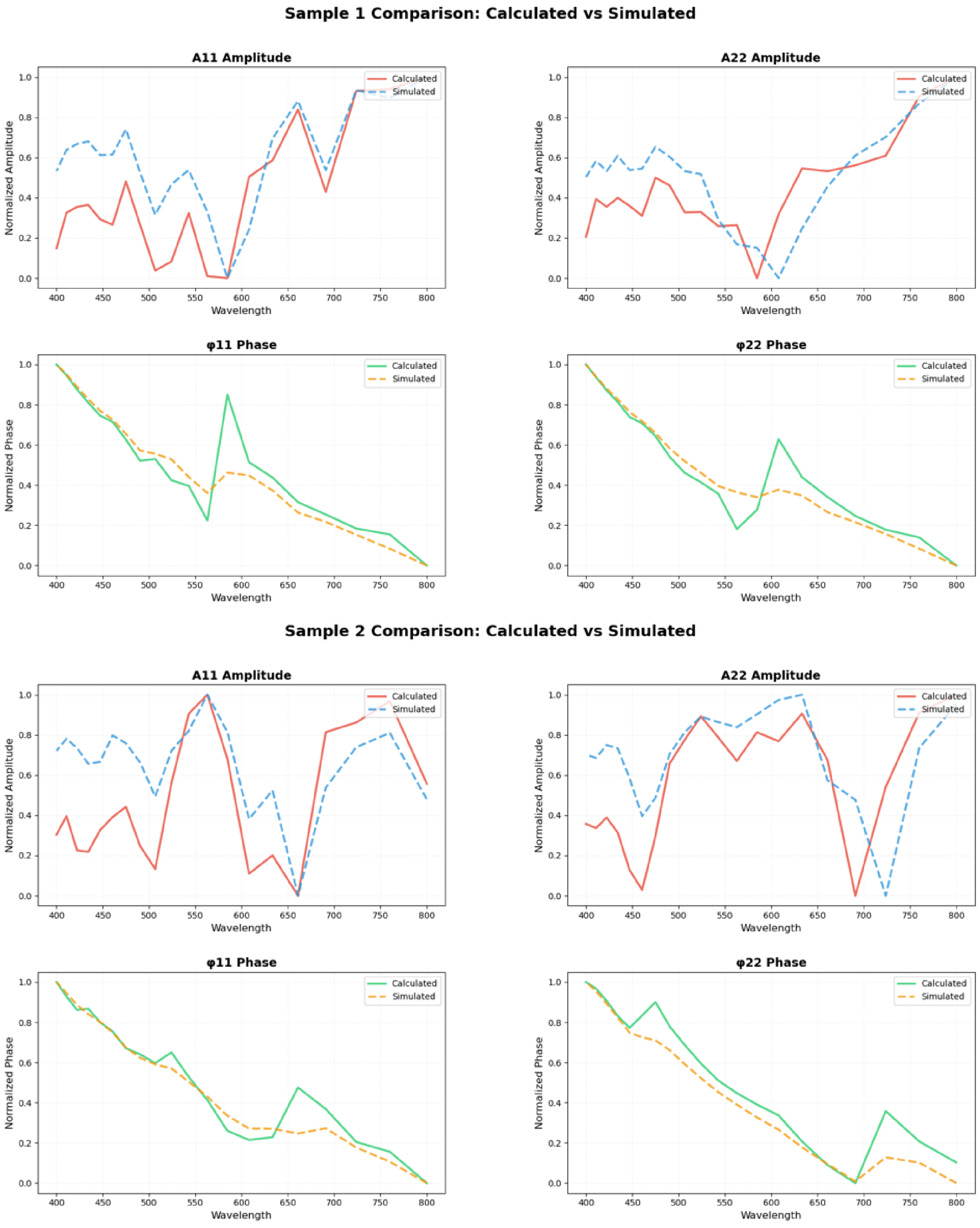

Figure S2: Wavelength-dependent Jones Matrix components generating through calculation (solid lines) and simulation (dashed lines).

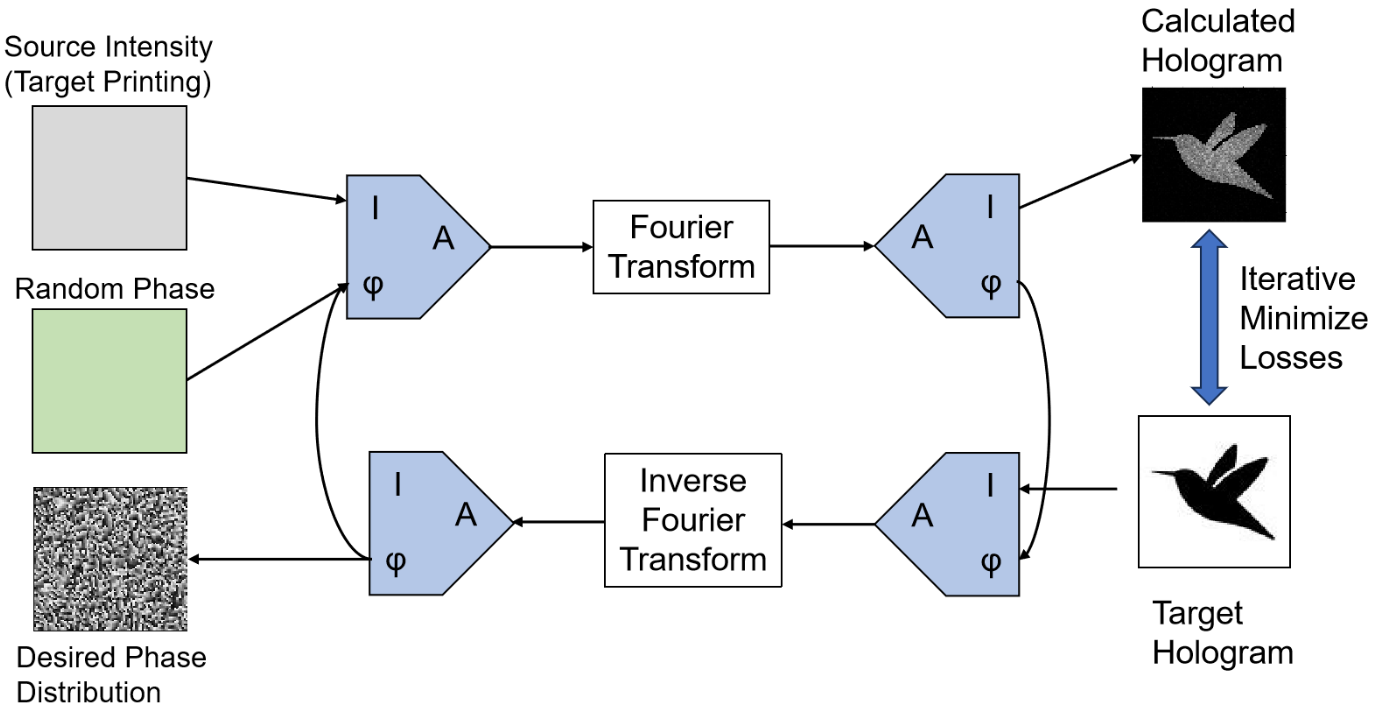

Figure S3: Schematic showing the GS algorithm used in our design phase.

4 Hyperparameter Tuning

The hyperparameter tuning of the model's pretraining (Table S2) reveals several key insights into optimizing its performance for metasurface inverse design. The goal is to minimize the loss, indicating better reconstruction of full-wavelength Jones matrices.

- Data Amount (Exp-001, 004, 005): A critical factor is the amount of data. While the initial tuning with 21.5 million data points (Exp-001, loss 0.0199) served as a baseline, reducing the data to 2.1 million (Exp-004) surprisingly yielded a lower loss (0.0079). This might suggest overfitting, since the amount of model parameters is much larger than the data size. However, the best performance was achieved with the largest dataset, 64.5 million points (Exp-005), resulting in the lowest loss of 0.0045. This confirms the general principle that more comprehensive data leads to a more robust and accurate model.

- Mask Type (Exp-001, 010-013): Although the mask type significantly influences pretraining loss, the variations mainly come from what percentage of Jones Matrices are covered, which strongly influence the difficulties to reconstruct.

- Batch Size (Exp-001, 002, 003): Decreasing the batch size from 128 to 32 (Exp-001 to Exp-003) gradually improved the loss from 0.0199 to 0.0189, but to run the model fast and efficiently, we kept using 128 batch size.

- Hidden Dimensions (Exp-001, 006): Increasing the hidden dimensions from 512 to 768 (Exp-006) slightly increased the loss (0.0201 compared to 0.0199). This suggests that 512 hidden dimensions might be sufficient since our input optical data are much simpler than normal images.

- Learning Rate and Decay Type (Exp-001, 008, 009): A higher base learning rate (2e-3 in Exp-008) resulted in a slightly worse loss (0.0208) compared to 5e-4 (Exp-001),

indicating that the original rate was closer to optimal, preventing overshooting during optimization. Regarding decay type, cosine decay (Exp-001) outperformed step decay (Exp-009), yielding a lower loss (0.0199 vs 0.0203). Cosine decay typically provides a smoother learning rate schedule, which can lead to better convergence.

- Relative Position Bias (Exp-001, 007): Including relative position bias (Exp-007) showed no change in loss (0.0199), suggesting it did not significantly impact performance under these specific pretraining conditions.

Table S2: Hyperparameter Tuning of Pretraining

Experiment ID	Batch Size	Data Amount	Hidden Dimensions	Relative Position Bias	LR(base/min/decay type)	Mask Type	Loss (epoch=100)
Exp-001	128	21526641	512	False	5e-4 / 5e-6 / cosine decay	1	0.0199
Exp-002	64	21526641	512	False	5e-4 / 5e-6 / cosine decay	1	0.0192
Exp-003	32	21526641	512	False	5e-4 / 5e-6 / cosine decay	1	0.0189
Exp-004	128	2152664	512	False	5e-4 / 5e-6 / cosine decay	1	0.0079
Exp-005	128	64579923	512	False	5e-4 / 5e-6 / cosine decay	1	0.0045
Exp-006	128	21526641	768	False	5e-4 / 5e-6 / cosine decay	1	0.0201
Exp-007	128	21526641	512	True	5e-4 / 5e-6 / cosine decay	1	0.0199
Exp-008	128	21526641	512	False	2e-3 / 2e-5 / cosine decay	1	0.0208
Exp-009	128	21526641	512	False	5e-4 / 5e-6 / step decay	1	0.0203
Exp-010	128	21526641	512	False	5e-4 / 5e-6 / cosine decay	2	0.0010
Exp-011	128	21526641	512	False	5e-4 / 5e-6 / cosine decay	3	0.0995
Exp-012	128	21526641	512	False	5e-4 / 5e-6 / cosine decay	4	0.0806
Exp-013	128	21526641	512	False	5e-4 / 5e-6 / cosine decay	5	0.0092

Notes:
1. During actual pretraining, the mask type is set to random, which would cause loss fluctuates and hard to evaluate. For clear comparison, the mask type is fixed mainly at Type1 with additional mask type tuning varying from 1-5.
2. Actual pretraining uses 64579923 data size, but to save time, above hyperparameter tuning process used one third (21526641) data size.

5 Analysis of the Impact of Model Parameter Count on Training and Inference

To address concerns related to model lightweighting and efficiency comparisons, we first clarify the rationale for parameter selection across different training phases, then analyze the performance of the original model compared to a lightweight variant.
For the pretraining phase, 60 million training samples were utilized. A parameter scale (37.7 million parameters) of the same order of magnitude as the data volume was selected

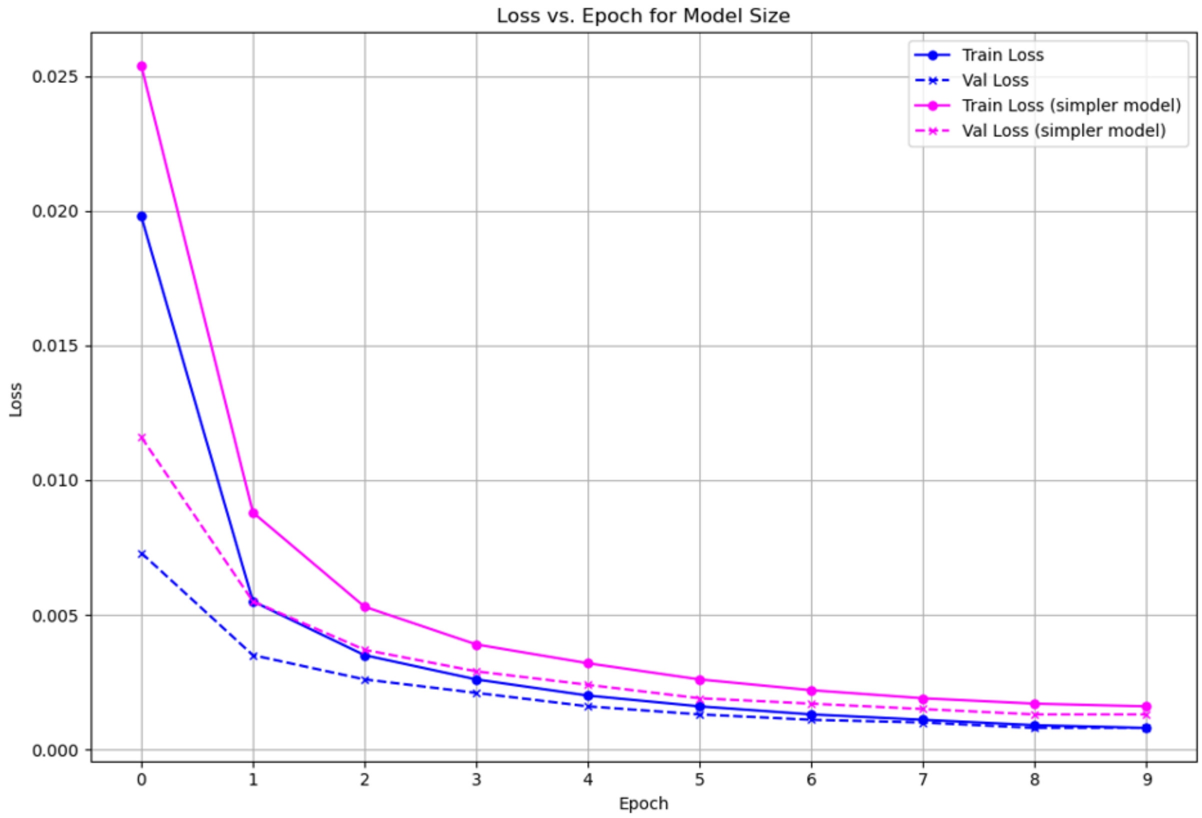

Figure S4: Loss vs Epoch learning curves of base model and simpler model.

to mitigate overfitting and underfitting. In contrast, the finetuning phase only used 1 million samples, rendering a lightweight model a feasible alternative for efficiency optimization.

The lightweight model was constructed by halving three key architectural components relative to the original model: embedding dimension (from 512 to 256), number of transformer blocks (depth, from 12 to 6), and number of attention heads (num_head, from 12 to 6). This modification reduced the number of learnable parameters from 37,705,542 to 4,749,270.

Table S3 summarizes the efficiency and performance metrics of the two models. In terms of training time, the lightweight model reduced the duration from 16 hours to 5 hours, but this reduction was not proportional to the decrease in parameter count. Two factors contributed to this discrepancy: (1) fixed training overheads (e.g., data loading, inherent costs of the PyTorch framework, and CUDA kernel launch expenses) that are independent of model parameter count; and (2) GPU utilization—while the significantly smaller lightweight model underutilized the computational capacity of four NVIDIA 4090 GPUs, the 37.7M-parameter original model fully leveraged the GPUs' capabilities.

In terms of inference efficiency, both models demonstrated comparable speed, processing 1000 samples in 6 seconds. However, the lightweight model exhibited inferior fitting performance: its training and validation losses were consistently higher than those of the original model across all training epochs, and it never achieved the low loss values attained by the original model (Figure S4). Notably, this comparison did not utilize pretrained weights. We emphasize that employing a full-scale model with the same architectural structure as the pretrained model (to leverage pretrained weights) could further enhance its efficiency and accuracy.

Table S3: Comparison of Model Architectures and Performance Metrics

Model Type	Embedding Dimension	Number of Transformer Blocks	Number of Attention Heads	Number of Parameters	Training Time	Inference Time (1000 samples)
Base model	512	12	12	37,705,542	16h31min20sec	6sec
Lightweighted model	256	6	6	4,749,270	5h01min17sec	6sec

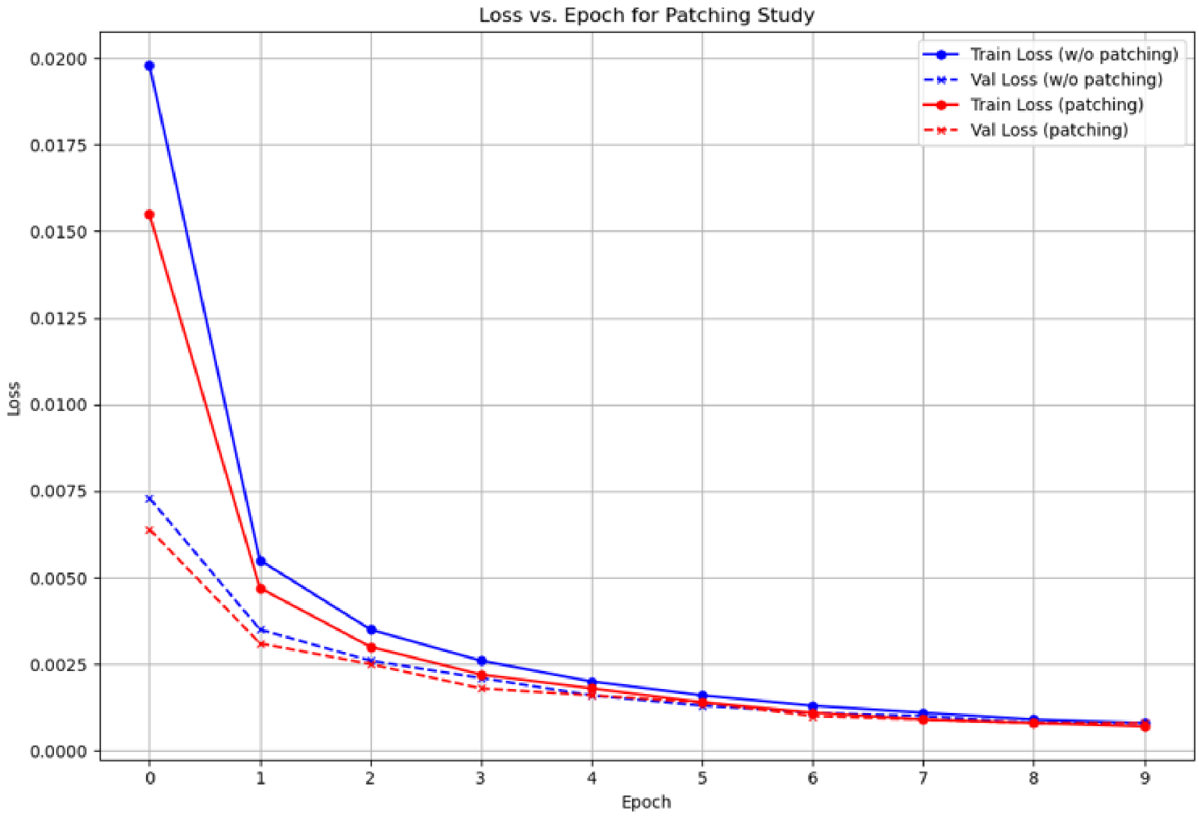

Figure S5: Loss vs Epoch learning curves of base model without patching and the model with patching.

6 Analysis of Patching Impact

To address concerns regarding the impact of patching in ViT and justify our approach of treating each physically significant matrix element as an individual token, we conducted a comparative analysis focusing on the fine-tuning workflow (noting pre-training is extremely time-consuming, making multiple pre-training runs impractical). We evaluated the learning curves of two models: one without patching (no-patching model) and another with a patch size of 3 (patching model), as presented in Figure S5. This figure plots training and validation loss against the number of epochs for both models.
As observed in Figure S5, the loss curves of the no-patching and patching models are remarkably similar throughout fine-tuning. In terms of learnable parameters, the no-patching model has 37,705,542 parameters, while the patching model has 37,654,342. Despite the slight parameter discrepancy, their comparable learning ability (evidenced by loss curves) indicates patching does not simplify the model—this stems from additional linear modules introduced by patching and dispatching processes.

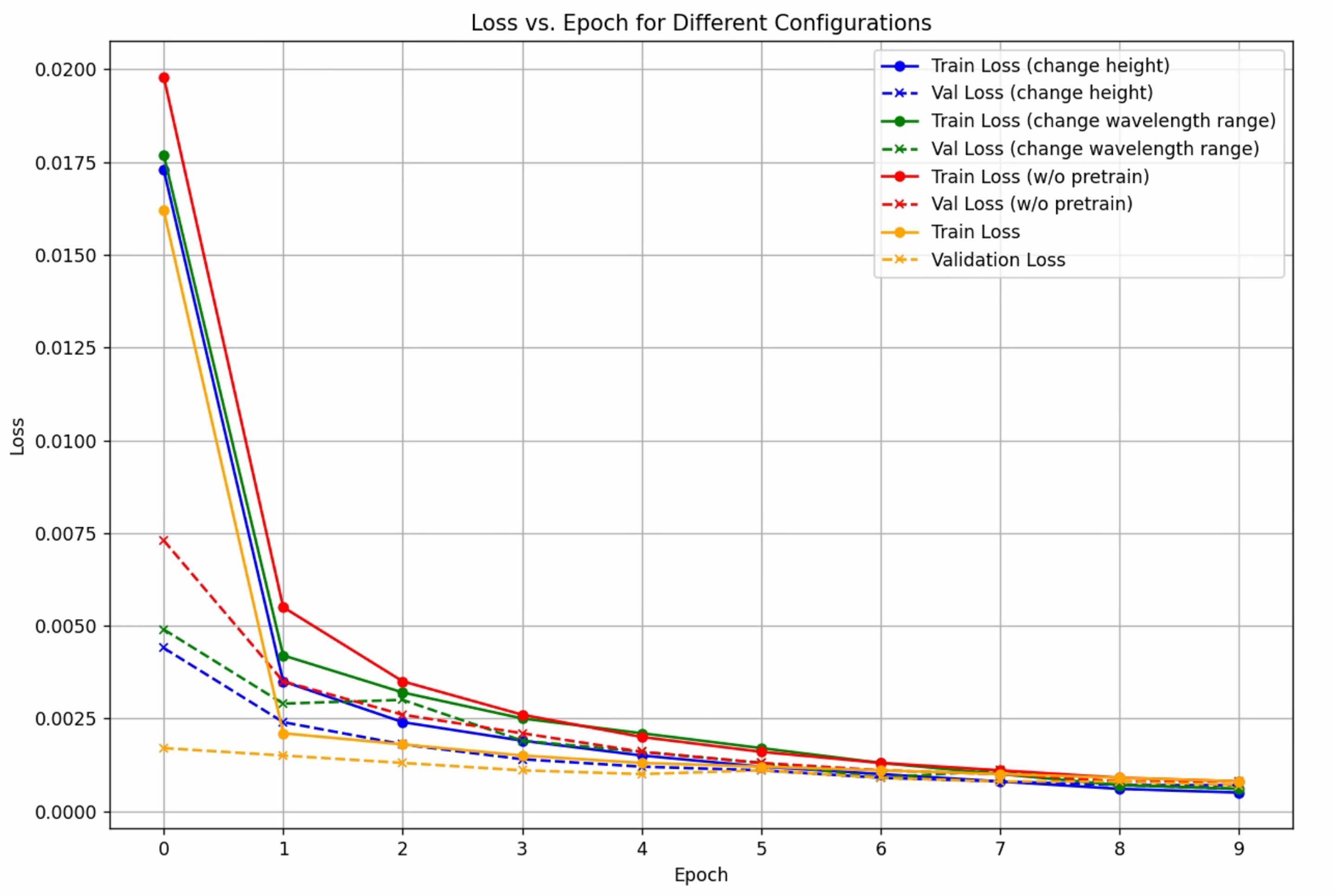

Figure S6: Loss vs Epoch for different configurations.

More importantly, our model is designed to learn fine-grained correlations (down to individual wavelengths and single Jones Matrix components) during pre-training. Patching would disrupt such precise dependencies; for example, when learning full Jones Matrices from single-wavelength data, patching might inappropriately mix signals across different wavelengths—undesirable for our physical task. Since we perform fine-tuning using pre-trained weights, maintaining structural consistency (i.e., omitting patching) throughout the workflow is well-founded.

7 Transfer Learning and Finetuning Process

In Figure S6, we presented learning curves of finetuning process under different conditions. Since the L1 losses decrease low enough at 10 epoch, we only present the loss change at 0-10 epoch in order to save computing time. First, the comparison between finetuning on pretrained weights and finetuning without pretraining indicates that they can both reach very low loss at epoch=10, demonstrating the efficiency and capability of our ViT model. However, at the first few epoches, finetuning on pretrained model experiences much more rapid training loss decay and holds very low validation loss, meaning it is at a ideal starting point which can save a lot of time on finetuning.
Secondly, the comparison between finetuning with the same data corpus and modified corpus shows the feasibility of convenient transfer learning. The curves in Figure S6 clearly show that their performances are comparable in the end (epoch = 10). Although their performances on training and validation sets underperforms default dataset at the begin-

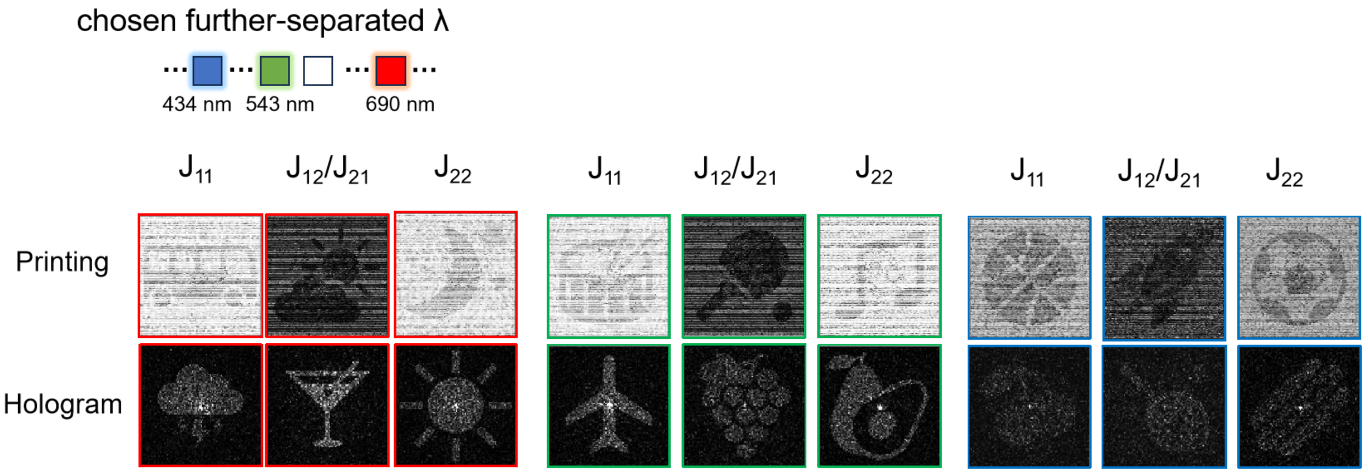

Figure S7: Illustration of 18-channel printing and hologram multiplexing accomplished at three further-separated wavelengths.

ning, they catch up very quickly at epoch=5, indicating the future transfer learning implementation would be quick and efficient. New-dataset-1 comes from the same basic data collection and data augmentation processes, and the only difference is the heights of all pillars change from 450 nm to 600 nm. The different part of new-dataset-2 is the wavelength range, which now covers the near-infrared range from 800 - 1200 nm. Notably, the transfer learning should also be implemented in pretrain model, so that we can perform the Jones Matrix reconstruction and the following workflow. Based on above finetuning performances, it is predictable that transfer learning on pretrain model would be also easy and feasible. The only difference is the output format, where finetuning model uses structural parameters but pretraining model uses full-scale Jones Matrices.

8 Further Optimization of Printings and Holograms Multiplexing

As we mentioned in the main text, due to the idealized design that cannot be fully reproduced, overlaps between three wavelength channels inevitably happened. To solve this issue, we first try selecting further separated wavelengths in order to reduce the crosstalk. It should be noted that the three wavelengths in the main text were randomly picked. As shown in Figure S7, we did obtain better performances with lower noise level in both printing and hologram images.

This passive strategy demonstrates our model can accomplish good print and hologram multiplexing as long as the design Jones Matrices are reasonable, but to be a generic model, a systematic method needs to be provided to optimize Jones Matrices design with complex dependencies. This issue arises because idealized design targets—for instance, demanding maximum amplitude at all operational wavelengths—are often not physically realizable. The model, when prompted with such targets, generates the closest possible full-scale Jones Matrices, yet still imperfect, leading to performance degradation. Therefore, we propose an iterative optimization framework. Our proposed solution is to refine the design target itself by using the pretrained model as a differentiable forward predictor within an optimization loop. The goal is to find an optimal and physically-aware set of design parameters G (the target Jones matrix components for each pixel) that minimizes a loss function

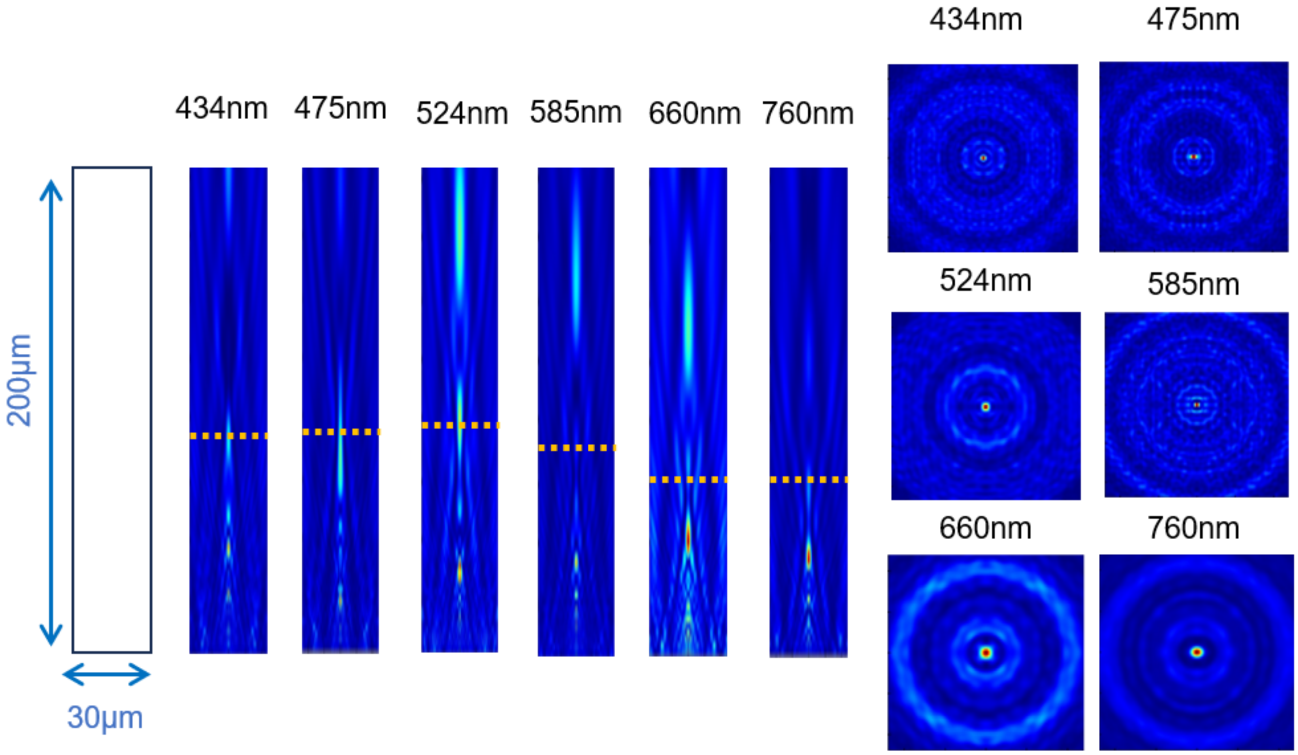

Figure S8: Left: Normalized electric field profiles within the x-z plane, indicative of the focusing length corresponding to each wavelength. Right: Normalized electric field profiles in the x-y plane illustrating the focusing performance at each wavelength.

$\mathcal{L}$, which quantifies the error between target Jones matrix and reconstructed Jones matrix (notice, it's the unmasked but not masked parts). This can be achieved using a gradient-based method:

$$G^{(t+1)} = G^{(t)} - \eta \nabla_G \mathcal{L}((f_{ViT}(G))) \tag{1}$$

Here, $G^{(t)}$ is the set of design parameters at iteration t, η is the learning rate, and f_{ViT} is our reconstruction model. By backpropagating the error gradient through the neural network, we can iteratively update the initial target G. This process nudges the design towards a self-consistent solution that is inherently compatible with the physical constraints learned by the model.

9 Further Optimization of Broadband Achromatic Metalens

In this section, we first discuss the metalens design merely using designed phase distribution and letting the pretrained model to reconstruct the amplitude parts. Compared with the strategy in the main text where all wavelength amplitude components are set to the maximum value, this strategy forces the model caring much more on the phase components. As shown in Figure S8, we observed better broadband achromatic performance. The focusing positions are less divergent comparing with Figure 5 in the main text. However, the light intensity (focusing efficiency) deteriorates indicating the loose of amplitude control during the reconstruction phase is a trade-off thing.

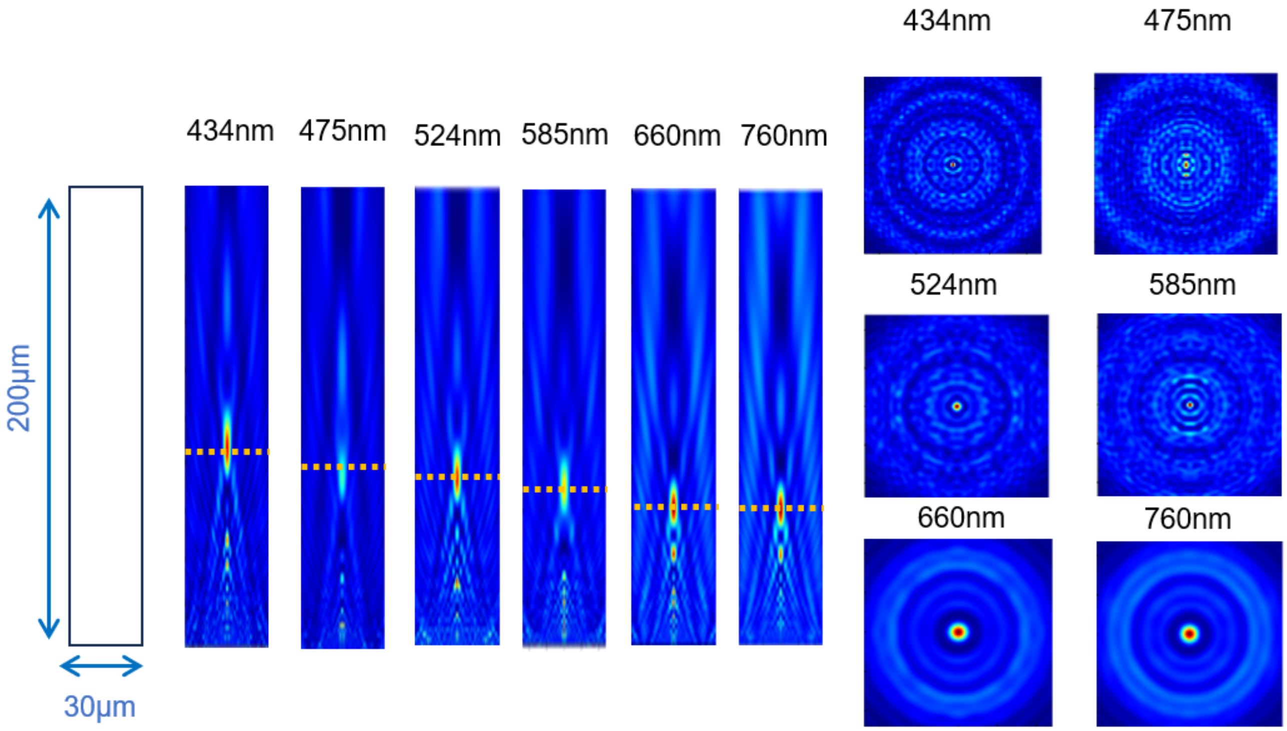

Figure S9: Results from Iterative Optimization. Left: Normalized electric field profiles within the x-z plane. Right: Normalized electric field profiles in the x-y plane.

Secondly, the optimization strategy we proposed above for multiplexing application can also be utilized in the metalens design. Revisiting Equation 1, the only difference lies in the meaning of $G^{(t)}$. Instead of designed Jones matrices at three wavelengths, here $G^{(t)}$ means the whole phase components at all wavelengths. Moreover, $G^{(t)}$ considers the amount of unit. Take a 200×200 array for example, the learnable parameters during the gradient descendant optimization should be $200 \times 200 \times 20 \times 3$, where 20 is the number of wavelength points and 3 is the components of phase in each wavelength.

Table S4: Parameters for Iterative Optimization Based on Gradient Descent

Parameter Category	Optimizer	Learning Rate	Number of Iterations	Learnable Matrix Shape
Values	torch.optim.Adam	0.001	500	[batch_size, x_size, y_size, wavelength_channel, JM_channel]

Using PyTorch and the parameters in Table S4, we established a gradient descent optimization workflow to obtain physics-constrained target Jones Matrices and generate valid size parameters. Substituting the size parameters from iterative optimization into FDTD simulations yielded field profiles at different wavelengths, presented in Figure S9.
In xz-plane field distributions, horizontal dashed lines indicate focusing lengths. Similar to the amplitude-masking approach in Figure S8, the iterative approach exhibits better achromatic performance than the original approach in Figure 5 (main text). Moreover, its light intensity (focusing efficiency) outperforms the amplitude-masking approach. However, the iterative process took over an hour even for a truncated metalens (64×128), revealing a trade-off between efficient one-shot inverse design and accurate iterative design.

As summarized in Table S5, the one-shot approach without amplitude constraints achieves the smallest focusing length standard deviation (7.2) and a reduced average focusing length (69.4), confirming enhanced chromatic convergence compared to the one-shot design with fixed amplitudes (std: 20.9, avg: 102.7). The iterative approach maintains a low standard deviation (9.3) with a comparable average focusing length (70.6) and superior focusing efficiency. These quantitative results further validate the trade-off between achromatic performance, focusing stability, and computational cost across the three inverse design strategies.

Table S5: Focusing Length Metrics Comparison Across Different Approaches

Approach	Focusing Length Average	Focusing Length Std
One-shot with amplitudes	102.7	20.9
One-shot w/o amplitudes	69.4	7.2
Iterative with amplitudes	70.6	9.3

10 Model Generalization Evaluation Comparing with Other Studies

In Table S6, we list representative studies focused on deep learning-assisted optical inverse design. To demonstrate the "genericity" of our model, this table highlights input/output types, tunability of wavelength/polarization, and whether our model can replicate their application scenarios. Here, "tunable" and "flexible" mean we can use arbitrary wavelengths (polarizations) or an arbitrary number of wavelengths (polarizations) as input.

11 Comparison between Data Retrieval and Deep-Learning Prediction

This section clarifies the added value of the neural network-based generator over database search/table lookup for inverse design based on target Jones matrices, and clarifies the role of the "matcher" step in the evaluation process.

First, the core advantage of deep learning lies in its generalization capability to unseen target Jones matrices, which distinguishes it from traditional data retrieval methods. Data retrieval merely retrieves existing entries from the database (60 million datasets) and cannot handle novel optical requirements. In contrast, our proposed neural network model learns the nonlinear physical mapping between Jones matrices and metasurface structure parameters, enabling interpolation and extrapolation for unseen targets. As the workflow shown in Figure S10, to verify this advantage, we conducted comparative experiments using 50 unseen samples, with results summarized in Table S7. Two masked proportions (19/20 and 10/20) were considered to simulate different levels of target novelty. For the masked proportion of 19/20, the Jones Matrix (JM) retrieval method achieved a mean absolute error (MAE) of 0.2308 and required an elapsed time of 1 h 11 min 32 sec, while our model's reconstruction method yielded a lower MAE of 0.0725 with a significantly shorter elapsed time of 20 sec. For the masked proportion of 10/20 (i.e., less novel targets), the retrieval method showed a slightly lower MAE of 0.0898 compared to the reconstruction method's 0.1114, but the retrieval still consumed much more time (1 h 29 min 20 sec vs. 20 sec). These results confirm that the neural network model not only learns internal patterns of data (rather than simple memorization) but also achieves higher efficiency.

Regarding the "matcher" step mentioned in the main text: the matcher is exclusively used for post-training evaluation, which follows a forward process (mapping from metasurface size parameters to Jones matrices). In this process, discretized 6-unit size parameters are used as keys for retrieval, which is more efficient than retrieving closest Jones matrices (a continuous distribution) in a backward process. However, the matcher still incurs high

Table S6: Comparison of Existing Metasurface Design Works and Compatibility with Our Model

Publication (Year)	Input	Output	Wavelength-Tunable Input?	Polarization-Tunable Input?	Model Compatibility	Reference
Nanophotonics 2020	Two continuous values (,)	1D grating parameters	No	No	Yes	[1]
Nanophotonics 2023	[1, 52] vector as transmission spectrum	2D image as structural info	No	No	Yes (with transfer learning to target wavelength range)	[2]
APL 2021	(x,y) coordinates on CIE gamut	Three size parameters	No	No	Yes	[3]
Adv Photon Res 2022	Full-scale S parameters (given wavelength range)	Binary 2D matrices (structure representation)	No	No	Yes (with transfer learning to target wavelength range)	[4]
ACS Photon 2022	S parameters (certain wavelength range)	Size parameters + 2D image	No	No	Yes	[5]
Nano Lett 2018	Transmission spectra	2D images	No	No	Yes	[6]
Laser Photon Rev 2024	Reflection spectra (certain wavelength)	Component-based structures	No	No	No	[7]
Optica 2025	Jones Matrix (4 components, single wavelength)	Structural parameters	No	No	Yes	[8]
Photon Res 2023	Jones Matrix (4 components, certain wavelength range)	Structural parameters	No	No	No (material mismatch: target uses metal + phase change materials)	[9]

Table S7: Comparison of MAE and Elapsed Time between JM Retrieval and Reconstruction (50 samples)

Masked Proportion	Retrieval MAE	Retrieval Elapsed Time	Reconstruction MAE	Reconstruction Elapsed Time
19/20	0.2308	1 h 11 min 32 sec	0.0725	20 sec
10/20	0.0898	1 h 29 min 20 sec	0.1114	20 sec

time costs when retrieving from millions of data entries for each unit in the metasurface. To quantify this, we compared the elapsed time and time complexity of the matcher (in raw and accelerated versions) and the neural network-based predictor using 27,648 samples, as shown in Table S8. The raw matcher required over 24 hours with a time complexity of $O(mn)$ (where m denotes the number of samples and n denotes the number of data entries in the database), while the accelerated matcher (leveraging numpy's parallel computing with C-language optimization) reduced the elapsed time to 3 h 12 min but retained the same $O(mn)$ complexity. In contrast, the predictor achieved an elapsed time of only 18 sec with a constant time complexity of $O(k)$ (where k denotes the number of parameters in

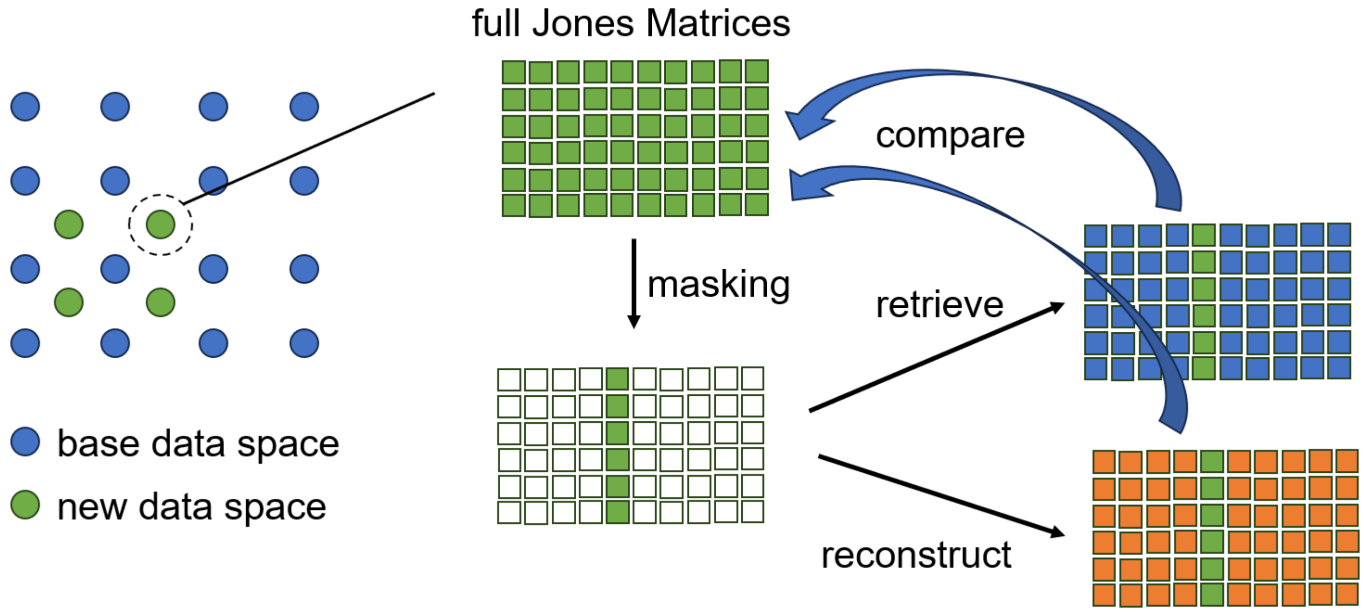

Figure S10: Schematic showing how to sample unseen data from data space, mask for retrieving and reconstructing, and compare with ground truth.

the neural network). This explains why the matcher was only used for early-stage evaluation, while the predictor served as the primary tool for high-efficiency evaluation.
In conclusion, the neural network-based approach not only significantly improves the efficiency of prediction and inference compared to data retrieval but also, more importantly, captures the hidden physical patterns in the data—an ability that traditional retrieval methods cannot achieve.

Table S8: Comparison of Elapsed Time and Time Complexity between Matcher and Predictor (27,648 samples)

Method	Elapsed Time	Time Complexity
Matcher (raw)	> 24 h	$O(mn)$
Matcher (accelerated)	3 h 12 min	$O(mn)$
Predictor (neural network)	18 sec	$O(k)$

References

[1] Jiaqi Jiang and Jonathan A Fan. Simulator-based training of generative neural networks for the inverse design of metasurfaces. *Nanophotonics*, 9(5):1059–1069, 2020.

[2] Zezhou Zhang, Chuanchuan Yang, Yifeng Qin, Hao Feng, Jiqiang Feng, and Hongbin Li. Diffusion probabilistic model based accurate and high-degree-of-freedom metasurface inverse design. *Nanophotonics*, 12(20):3871–3881, 2023.

[3] Nathan Bryn Roberts and Mehdi Keshavarz Hedayati. A deep learning approach to the forward prediction and inverse design of plasmonic metasurface structural color. *Applied Physics Letters*, 119(6), 2021.

[4] Mehdi Kiani, Jalal Kiani, and Mahsa Zolfaghari. Conditional generative adversarial networks for inverse design of multifunctional metasurfaces. *Advanced Photonics Research*, 3(11):2200110, 2022.

[5] Ibrahim Tanriover, Doksoo Lee, Wei Chen, and Koray Aydin. Deep generative modeling and inverse design of manufacturable free-form dielectric metasurfaces. *ACS Photonics*, 10(4):875–883, 2022.

[6] Zhaocheng Liu, Dayu Zhu, Sean P Rodrigues, Kyu-Tae Lee, and Wenshan Cai. Generative model for the inverse design of metasurfaces. *Nano letters*, 18(10):6570–6576, 2018.

[7] Yuetian Jia, Zhixiang Fan, Chao Qian, Philipp del Hougne, and Hongsheng Chen. Dynamic inverse design of broadband metasurfaces with synthetical neural networks. *Laser & Photonics Reviews*, 18(10):2400063, 2024.

[8] Haoran Wei, Xiaoyong He, and Wenhan Cao. Spin-multiplexed metasurface inverse-design based on a bi-directional deep neural network for terahertz wavefront control. *Optica*, 12(4):505–517, 2025.

[9] Zhi-Dan Lei, Yi-Duo Xu, Cheng Lei, Yan Zhao, and Du Wang. Dynamic multifunctional metasurfaces: an inverse design deep learning approach. *Photonics Research*, 12(1):123–133, 2023.
